# Supplementary figures and images for: HIV-1 exploits LBPA-dependent intraepithelial trafficking for productive infection of human intestinal mucosa
Source: PLoS Pathog. 2024 Dec 27;20(12):e1012714. doi: 10.1371/journal.ppat.1012714 (PMC11676502; doi:10.1371/journal.ppat.1012714)

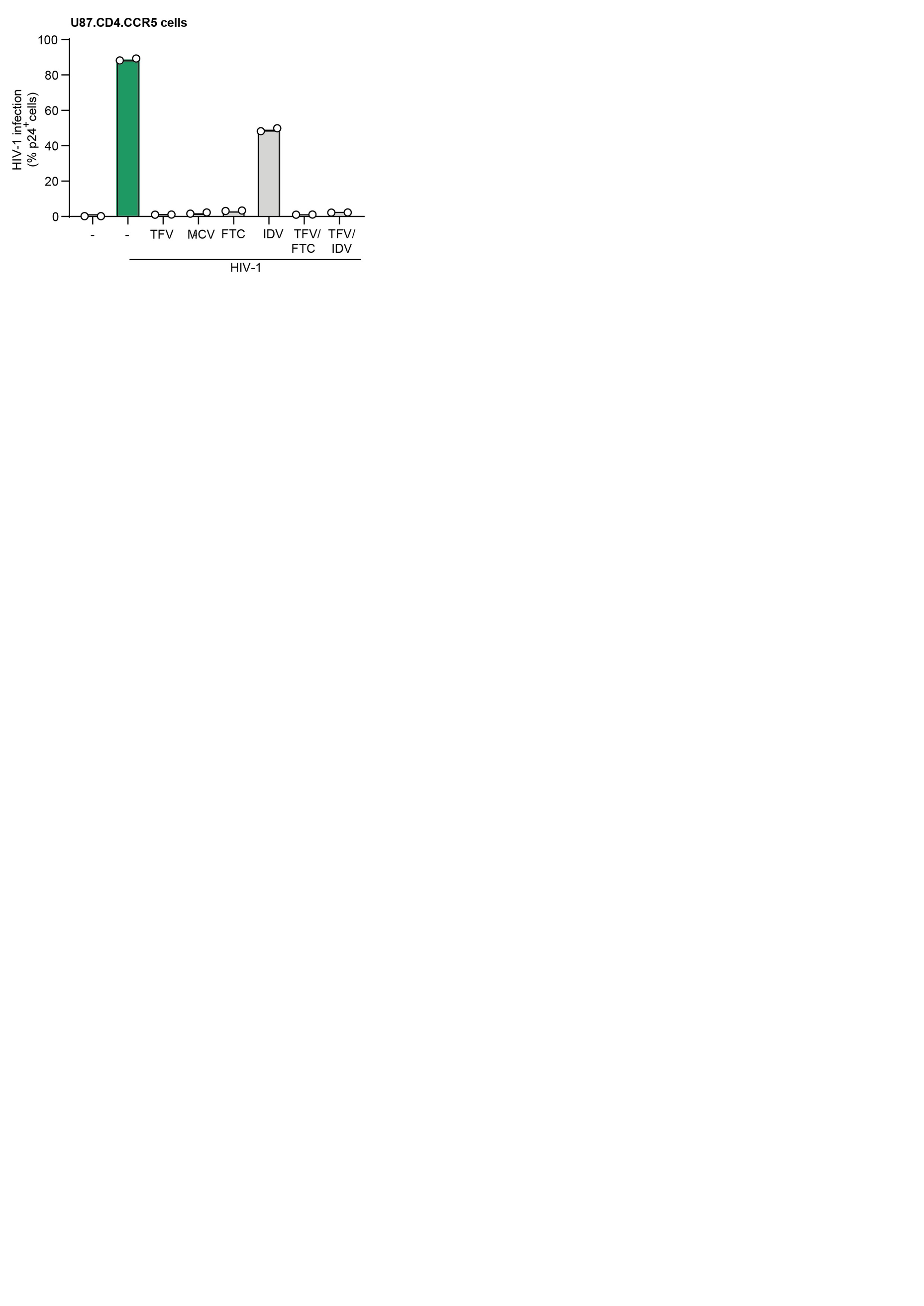

Supplement: S1 Fig — Productive HIV-1 infection of U87.CD4.CCR5 cell line, determined by intracellular p24 staining by flow cytometry analyses. U87.CD4.CCR5 cells were pre-treated with nucleotide reverse-transcriptase inhibitor Tenofovir (TFV, 50 μM), CCR5-antagonist Maraviroc (MCV, 30 μM), nucleoside reverse-transcriptase inhibitor Emtricitabine (FTC, 1 μM), protease inhibitor Indinavir (IDV,1 μM), or a combination of TFV (25 μM) with either FTC (0.2 μM) or IDV (0.2 μM) for 2h or left untreated prior to exposure to HIV-1 NL4.3BaL for 72h. Open circles represent n = 2 biological replicates. (TIF) [file ppat.1012714.s001.tif]

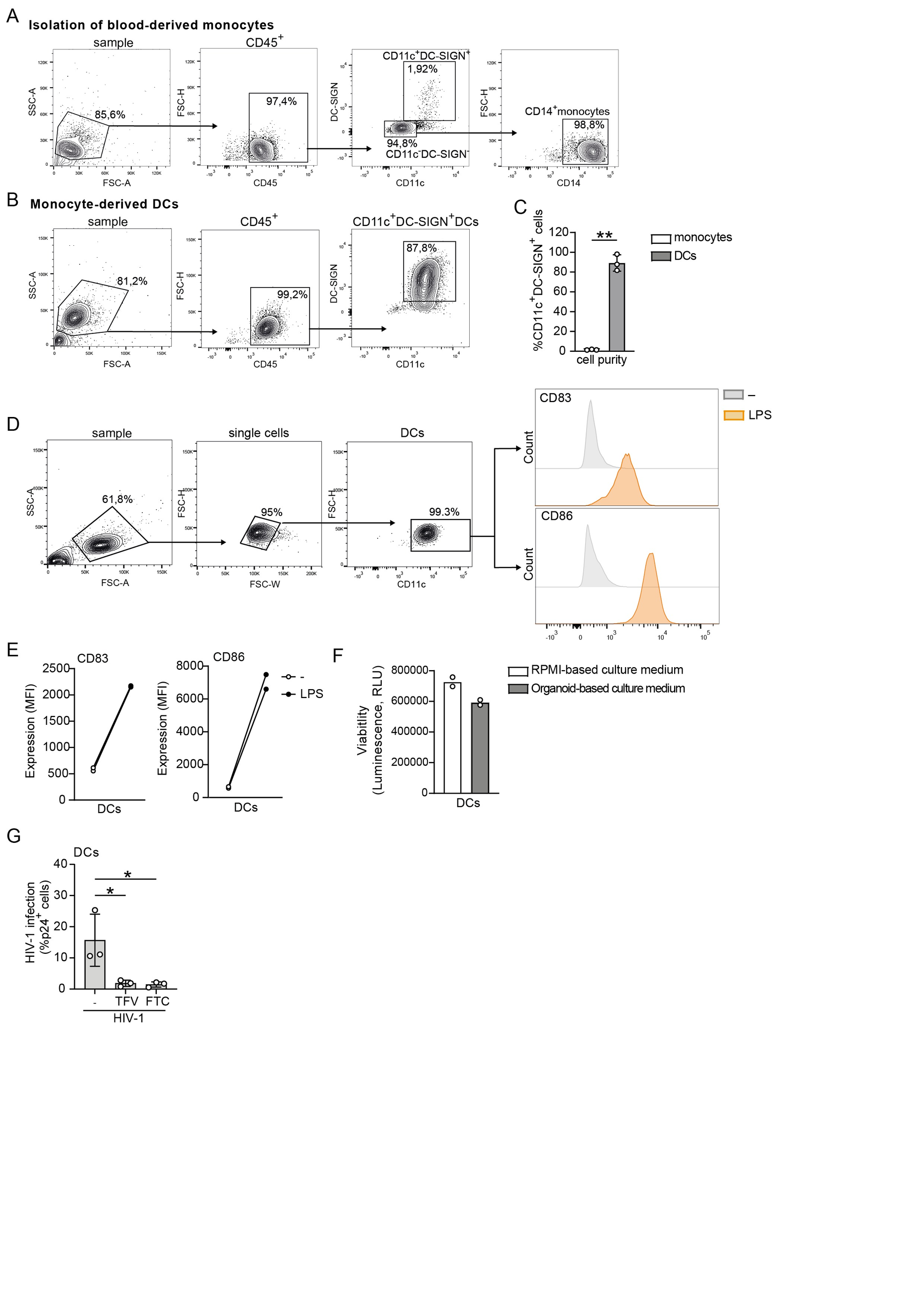

Supplement: S2 Fig — (A-C) Purity of monocyte-derived DC differentiation was routinely determined by surface staining for nucleated hematopoietic cell marker CD45, monocyte marker CD14 and DC markers CD11c and DC-SIGN by flow cytometry analyses. Representative flow cytometry plots of the (A) phenotypic characterization of affinity purified CD45+CD11c-DC-SIGN-CD14+ human monocytes from PBMCs by positive immunomagnetic selection using CD14 Microbeads and (B) differentiation of the isolated monocytes to CD45+CD11c+DC-SIGN+ DCs (on day 4 of differentiation as used to establish the IEDC co-cultures). (C) Quantification of purity of monocyte-derived CD11c+DC-SIGN+ DCs prior to establishment of IEDC co-cultures. Data are mean ± SD of n = 3 donors, Student’s paired t test. **p<0.01. (D,E) Maturation of monocyte-derived DCs was determined by surface staining for canonical activation DC markers CD83 and CD86 by flow cytometry analyses. (D) Representative flow cytometry plots and histograms of untreated DCs (as used to establish the IEDC co-cultures) or DCs treated with lipopolysaccharide (LPS,10 ng/mL) for 48h. LPS-treated DCs displayed a mature phenotype, in contrast to untreated DCs, as evident by increased expression of maturation markers CD83 and CD86. (E) quantification of flow cytometry data. n = 2 donors. (F) Viability of DCs cultured in different media formulations, determined by ATP-Based CellTiter-Glo luminescent cell viability assay. DCs were either cultured 24h in standard RPMI-based DC culture medium (i.e. RPMI 1640 supplemented with 10% FCS, 10 U/ml and 10 μg/ml, penicillin/streptomycin respectively, 2 mM L-glutamine, 500 U/mL IL-4 and 800 U/mL GM-CSF) or standard organoid differentiation medium supplemented with GM-CSF. Data is mean of n = 2 donors (RLU, relative light units). (G) Productive HIV-1 infection of DCs, determined by intracellular p24 staining by flow cytometry analyses. DCs were pre-treated with nucleotide reverse-transcriptase inhibitor Tenofovir (TFV, 25 μM) or nucle [file ppat.1012714.s002.tif]

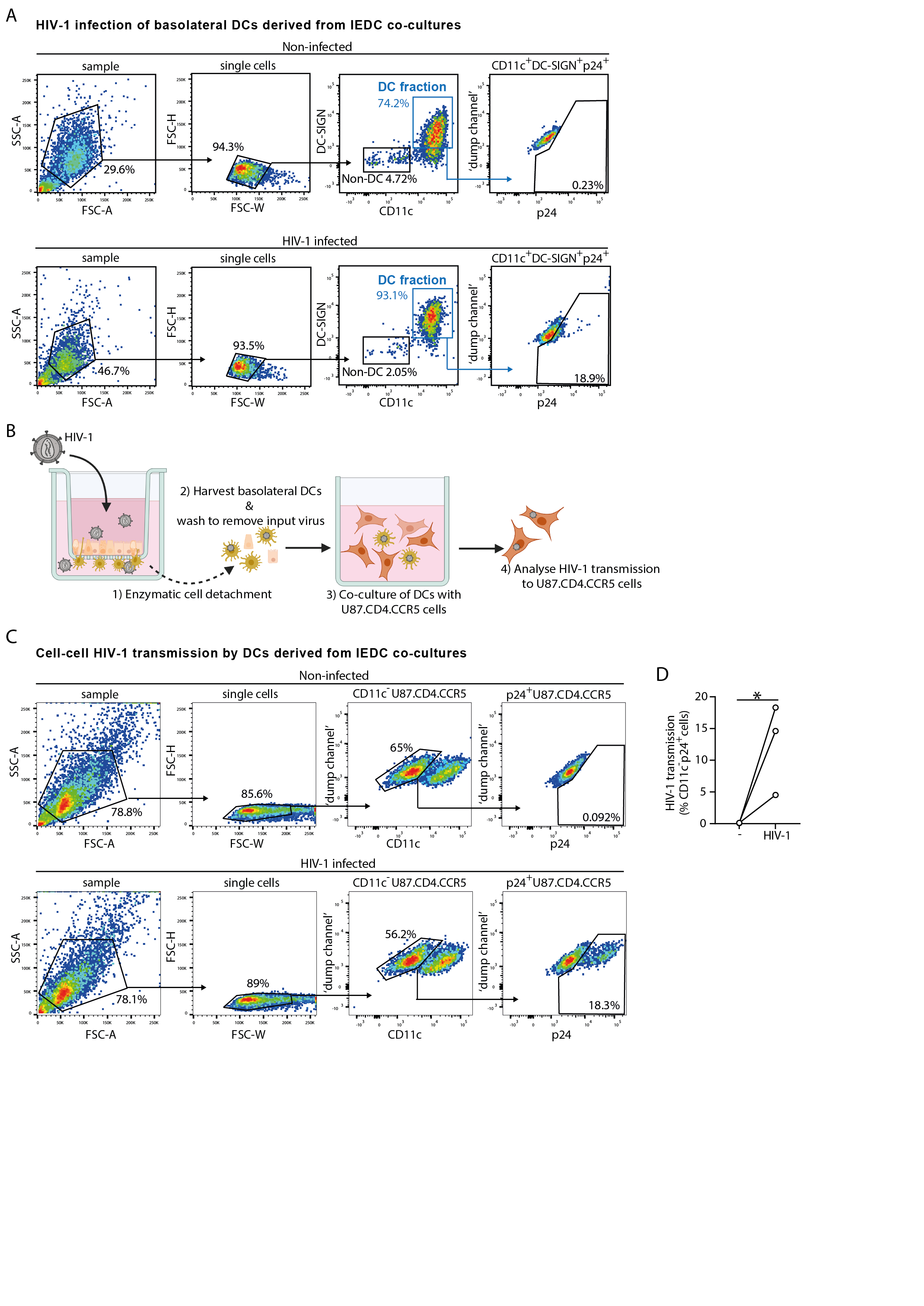

Supplement: S3 Fig — (A) Gating strategy to determine HIV-1 infection of detached basolateral CD11c+DC-SIGN+ DCs derived from non-infected versus HIV-1 exposed IEDC co-cultures using multiparameter intracellular p24 staining by flow cytometer analyses. (B) Schematic representation of enzymatic cell detachment treatment of the basolateral compartment from IEDC co-cultures to determine DC-mediated HIV-1 transmission via cell-cell contact to U87.CD4.CCR5 cell line. (C-D) IEDC co-culture models were apically exposed to HIV-1 NL4.3BaL for 72h or left non-infected, followed by Accutase-mediated cell detachment treatment to harvest the basolateral DCs. Basolateral DCs were subsequently washed to remove input virus and co-cultured with HIV-1 permissive U87.CD4.CCR5 target cells for 96h. DC marker CD11c was used to exclude single DCs and DC-U87 conjugates from flow cytometry analyses [12]. HIV-1 infection of U87.CD4.CCR5 in co-culture with DCs was thereby defined as CD11c-p24+cells and is representative of cell-cell HIV-1 transmission by DCs from IEDC co-cultures, determined by multiparameter intracellular p24 staining by flow cytometer analyses. (C) Representative flow cytometry plots and (D) quantification of flow cytometry data. n = 3 donors. Ratio paired t-test, *p<0.05. (TIF) [file ppat.1012714.s003.tif]

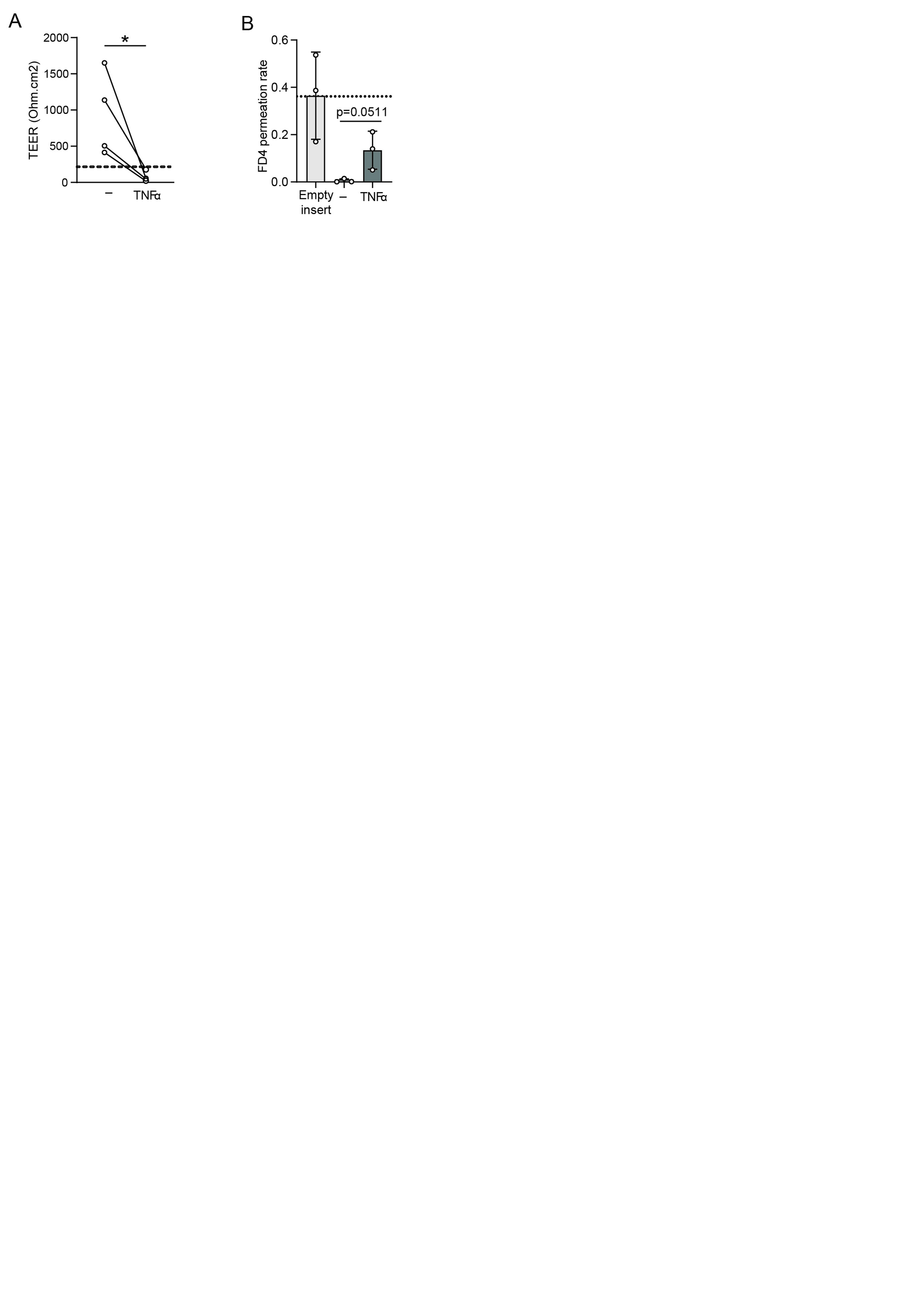

Supplement: S4 Fig — (A,B) TNF-α treatment (400 ng/mL for 96h) compromises epithelial barrier integrity and paracellular permeability in IE cultures as compared to untreated control. (A) Integrity of IE cultures determined by trans-epithelial electrical resistance (TEER) measurements. TEER value ≥200 Ohm.cm2 (dashed line) indicative of a confluent monolayer. n = 4 donors, Student’s paired t-test, *p<0.05. (B) Paracellular permeability of empty inserts, untreated or TNF-α treated IE cultures, determined by 4 kDa FITC-conjugated dextran (FD4) permeation rate of monolayers at 4h post-FD4 addition. Permeability is expressed as FD4 permeation rate: FD4 basolateralt = 4(μg)/FD4 apicalt = 0(μg). Dashed line (0.36) indicates the average of FD4 permeation rate from empty inserts. Data are mean ± SD of n = 3 donors, Student’s paired t-test. (TIF) [file ppat.1012714.s004.tif]

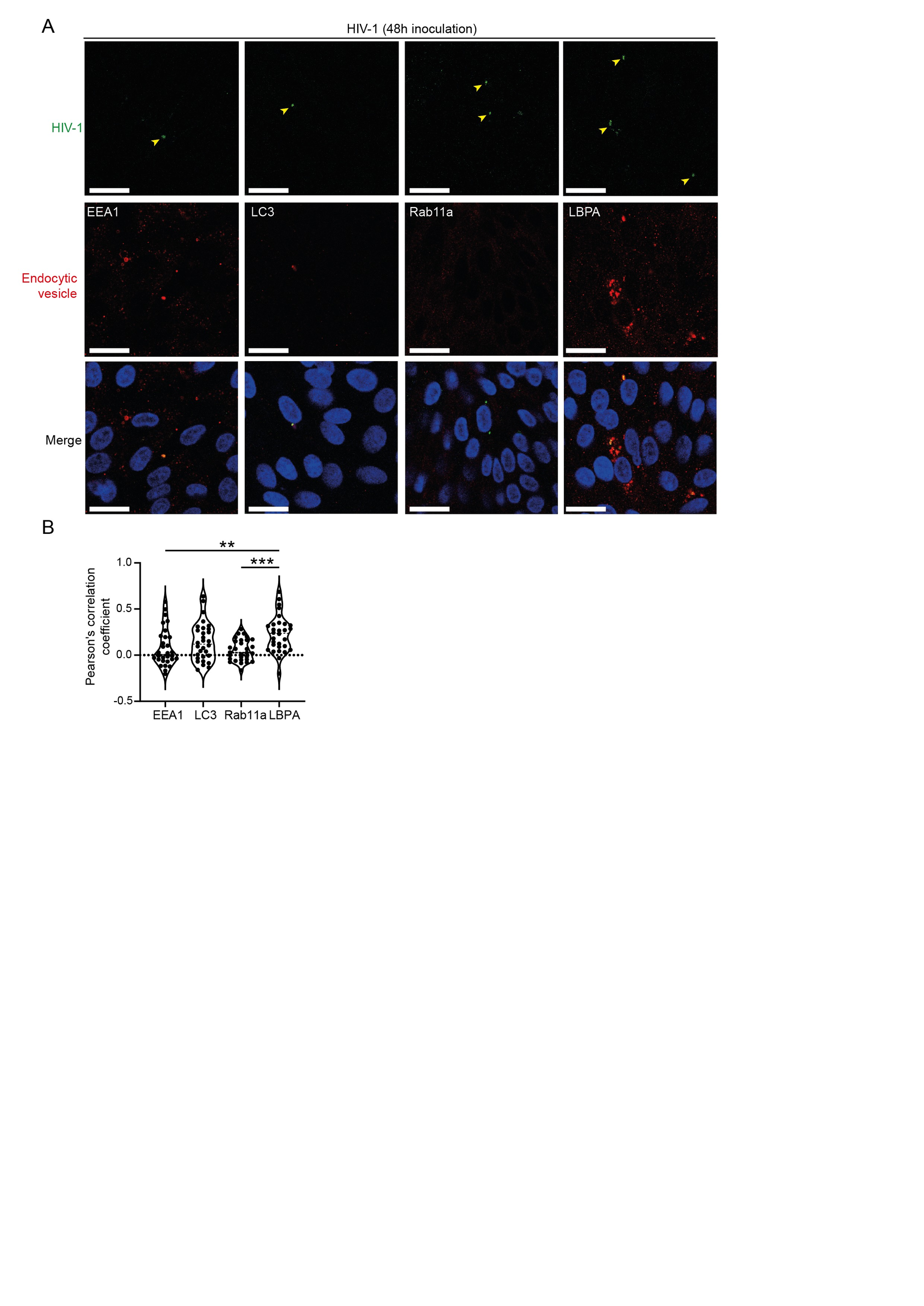

Supplement: S5 Fig — (A) Colocalization of endocytic vesicle markers in IE cultures upon 48h exposure to HIV-1 Gag-iGFP by confocal microscopy analyses. Endocytic vesicle markers (EEA1, LC3, Rab11a, LBPA, red), HIV-1 (green, filled arrowheads). Scale bar = 20 micron, representative of n = 3 donors. (B) Pearson’s correlation coefficients for colocalization analyses between each endocytic vesicle marker and HIV-1, n = 3 donors, Two-way ANOVA, **p<0.01, ***p<0.001. (TIF) [file ppat.1012714.s005.tif]
