# Supplementary material for: HIV-1 exploits LBPA-dependent intraepithelial trafficking for productive infection of human intestinal mucosa
Source: PLoS Pathog. 2024 Dec 27;20(12):e1012714. doi: 10.1371/journal.ppat.1012714 (PMC11676502; doi:10.1371/journal.ppat.1012714)
Supplement: Attachment — Submitted filename: Rader et al. response to reviewers.pdf [file ppat.1012714.s007.pdf]

Response to reviewer 1

Summary: The manuscript "HIV-1 exploits LBPA-dependent intraepithelial trafficking for productive infection of human intestinal mucosa" by Rader et al. describes a mechanism by which epithelial cells transmit HIV to underlying target cells. The authors establish a polarized immune-organoid model – consisting of apical intestinal epithelial cells and basolateral monocyte-derived cells - to demonstrate a role for LBPA-rich endocytic vesicles in mediating transcellular transport of HIV across a polarized epithelial barrier. The authors demonstrate that the addition of DCs to the epithelial model amplifies HIV-1 transmission, that pre-treatment of epithelial cells with tenofovir does not reduce their ability to transmit virus, and that treatment of the immune-organoid culture with anti-LBPA reduces transmission. The studies described in this manuscript describe a novel mechanism in which epithelial endosomes contribute to transcellular transport and may inform the design of novel inhibitors of epithelial transcytosis.

The work presented in this manuscript convincingly presents several interesting observations and is clearly representative of significant effort. It is unclear however, whether the focus of this work should be the utility of the IEDC model or the role of LBPA-mediated epithelial transcytosis in seeding/amplifying intestinal infection. The authors describe the results from several experiments that address these topics but do not sufficiently explore either individually. Although the results and insights conveyed by this manuscript are of interest and potential importance, the manuscript does not yet fully support the conclusions drawn by the authors.

Major comments:

1. The authors state that "exposure of intestinal epithelium to HIV-1 results in viral internalization in a manner that is impervious to antiretroviral treatment", in the abstract and at other points throughout the manuscript. Although the authors demonstrate that treatment of epithelial cells with tenofovir allows for subsequent virus amplification in U87.CD4.CCR5 cells, the authors utilized only a reverse transcriptase inhibitor. Did the authors use other classes of antiretrovirals - was internalization/transmission still 'impervious? Could the authors use single-round viruses to further explore the transmission potential of epithelial cells in the context of antiretrovirals? Using a single-round virus, increased transmission potential from antiretroviral pre-treated epithelial cells as compared to treated lamina propria cells or dendritic cells would more convincingly demonstrate the antiviral-resistant transmission potential of epithelial cells.

- As requested, we now show that HIV-1 transmission by human primary intestinal epithelial cells is not only impervious to prophylactic treatment with nucleotide analog reverse transcriptase inhibitor Tenofovir (TFV), but also to prophylactic treatment with either CCR5 antagonist Maraviroc (MCV) or nucleoside analog reverse transcriptase inhibitor Emtricitabine (FTC) or protease inhibitor Indinavir (IDV). We have included these new data (Revised manuscript: Figure 2F,G ; Supplementary Figure S1 and S2G) and clarified the findings in the revised manuscript (Revised manuscript: lines 122-146). As suggested, we have also investigated the ability of antiretrovirals to interfere with the transmission of single-round immature HIV-1 particles by epithelial cells and DCs. To this end, human primary intestinal epithelial cells or human primary DCs were pre-treated with protease inhibitor Indinavir (IDV) – which results in the production of immature viral particles - in the presence or absence of reverse transcriptase inhibitor Tenofovir (TFV) followed by HIV-1 inoculation (*Rebuttal Figure 1, please see below*). Seminal reports (PMID: 20364151, 33846309, 23271952, 10721995, 27919079) have shown that human primary dendritic cells are susceptible to HIV-1 infection and are able to transmit HIV-1 in a replication-dependent (i.e *cis*-infection of HIV-1) via fusogenic receptors such as CD4, CCR5, CXCR4 as well in a replication-independent manner (i.e. *trans*-infection of HIV-1) via C-type lectin receptors such as DC-SIGN and Siglec-1, with the latter not being inhibited by antiretroviral drugs. Intestinal epithelial cells, on the other hand, were shown to be able to transfer

HIV-1 to CD4+ cells in a replication-independent manner (PMID: 11821899, PMID: 8986739). In concordance with previous studies, we show that prophylactic IDV treatment reduced DC-mediated HIV-1 transmission but remained ineffective in epithelial cell-mediated HIV-1 transmission. Co-treatment with TFV decreased HIV-1 transmission by IDV-treated DCs, however was still unable to limit transmission by IDV-treated epithelial cells (*Rebuttal Figure 1, please see below*). These results further underscore the sole ability of intestinal epithelial cells to transmit infectious HIV-1 without *de novo* viral replication (i.e *trans*-infection of HIV-1), and thereby invulnerable to treatment with antiretrovirals.

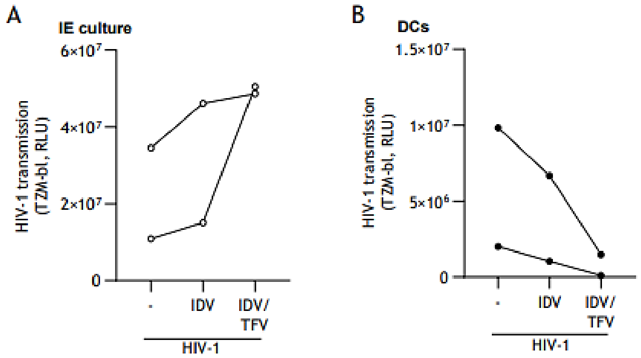

Rebuttal figure 1. HIV-1 transmission by IE cultures is independent of viral replication. (**A**) Intestinal epithelium (IE) cultures were pre-treated with protease inhibitor Indinavir (IDV, 1 µM), or a combination of IDV (1 µM) with nucleotide reverse-transcriptase inhibitor Tenofovir (TFV, 25 µM) for 1h or left untreated prior to 72h exposure to HIV-1 NL4.3BaL. Subsequently, basolateral supernatant from virus-exposed IE cultures was collected, and co-cultured with HIV-1 permissive TZM-bl cell line. Pre-treatment with either IDV or IDV/TFV co-treatment did not suppress IE-mediated HIV-1 transmission to the TZM-bl reporter cell line. (**B**) Dendritic cells (DCs) were pre-treated with Indinavir (IDV, 1 µM), or a combination of IDV (1 µM) with TFV (25 µM) for 1h or left untreated prior to 48h exposure to HIV-1 NL4.3BaL. Virus-exposed DC were subsequently washed to remove input virus and co-cultured with TZM-bl cell line. Pre-treatment with antiretroviral therapeutics limit DC-mediated HIV-1 transmission to the TZM-bl cell line. (**A,B**) HIV-1 transmission by IE cultures (**A**) or by DCs (**B**) was determined by luciferase activity (RLU) 48h post co-culture in TZM-bl cell line. Open and closed circles represent individual *n* = 2 gut or blood donors, respectively.

2. The authors describe that the basolateral cells their IEDC co-cultures (Figure 2 and Lines 134-151) are predominantly CD11c+ cells but they do not confirm that these cells are DCs by alternate methods nor do they describe the remaining (CD11c-) cell populations or their contribution to HIV amplification in their model. Although the authors suggest that their methodology produces "high-purity immature DCs expressing canonical DC markers" (Line 340 and Figure S1) independent of adhesion, they do not appear to establish that their post-adhesion, basolateral-associated monocytes are differentiated DCs. Indeed only 75% of their basolateral detached DCs (Figure 2F) are CD11c+, which itself is not an exclusive DC marker. A more detailed phenotypic description of the putative DCs in Figure 2F and of the contribution of the non-DC compartment to transmission in the IEDC model would strengthen the proposed contributions of DCs to viral transmission in the IEDC model.

- As requested, we have now included the extended phenotypic characterization of isolated blood-derived monocytes and subsequently differentiation to monocyte-derived DCs using prototypical cellular markers (PMID: 12351396, 29313948). In line with previous reports (PMID: 33637808, 27919079), the protocols employed in our manuscript routinely result in highly pure monocytes (~96% $CD45^+CD14^+$ monocytes) and canonical monocyte-derived DCs (~90% $CD45^+CD11c^+$DC-$SIGN^+$ DCs). We have included these data (Revised manuscript: Supplementary Figure 2A,B,C) and clarified the findings in the revised manuscript (Revised manuscript: lines 393-401; 918-927). In Figure 2F (*initial version of the manuscript*), we aimed to retrieve the adherent basolateral

attached DCs for downstream analyses by subjecting the basolateral compartment to enzymatic cell detachment (accutase-based) treatment. The proteolytic and collagenolytic enzyme activity of accutase enables the gentle detachment of basolateral DCs (~85% of CD45$^+$CD11c$^+$DC-SIGN$^+$ DC fraction) accompanied by co-detachment of a subpopulation of non-immune epithelial cells (~14% CD45$^-$ cell fraction). The CD11c$^+$ fraction thereby represents the detached DC fraction and the CD11c$^-$ fraction represents the detached non-DC fraction. Our data show that the retrieved DC compartment (CD11c$^+$DC-SIGN$^+$ cell fraction) from virus-exposed IEDC co-cultures, are readily infected with HIV-1, in contrast to the retrieved non-DC compartment (CD11c$^-$DC-SIGN$^-$ cell fraction), which further strengthens the role for subepithelial DCs in amplifying HIV-1 transmission in the IEDC model. We have included these additional data (Revised manuscript: Figure 3F, H, I and Supplementary Figure S3A) and clarified the findings in the revised manuscript (Revised manuscript: lines 517-531; 834-843).

3. Although the IEDC model adds biological relevance, its use in Figure 5F-H muddles the interpretation that LBPA-mediated epithelial transcellular transport serves as an important source of viral transmission during HIV acquisition across an epithelial barrier. This mechanism is addressed more succinctly in Figure S4 – without the addition of DCs - though more replicates are needed to convincingly demonstrate significance. Alternately, did the authors try a step-wise approach in their method development – treating epithelial cells with anti-LBPA and HIV (followed by wash-out), prior to DC attachment?

- Neutralization of LBPA-mediated endosomal route results in a significant reduction of HIV-1 transmission mediated by IE cultures (Revised manuscript: Figure 7C *p*=0.0196) as well as by IEDC co-cultures (Revised manuscript: Figure 7F *p*=0.0189). The suppressive effect of anti-LPBA across not only the epithelial-based organoid model, but also across the more advanced immune-competent organoid co-culture model thus substantiate the therapeutic potential of LBPA-targeting strategies to intervene in subepithelial mucosal HIV-1 propagation. Furthermore, and as suggested, we now show that anti-LBPA treatment via the step-wise approach (*Rebuttal Figure 2, see below*) also resulted in decreased HIV-1 transmission by IEDC co-cultures, which supports the role for LBPA-mediated epithelial transcellular HIV-1 route to replicate within intestinal mucosa.

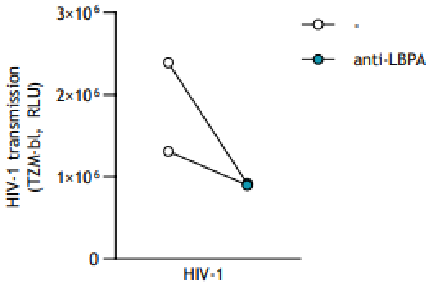

Rebuttal figure 2. Prophylactic anti-LBPA treatment in HIV-exposed IE cultures prior to basolateral DC attachment reduced HIV-1 transmission by IEDC co-culture. IE cultures were apically pre-treated with either anti-LBPA antibody (15 µg/ml, green circles) or left untreated (open circles) for 16h, followed by exposure to HIV-1 NL4.3BaL for 24h. Subsequently, apical and basolateral compartments were washed to remove input virus and DCs were seeded onto the basolateral side of the insert membrane in the IE cultures for 2h to generate the IEDC co-cultures. After 48h incubation, basolateral supernatant from IEDC co-cultures was harvested and co-cultured with the TZM-bl reporter cell line for 48h. HIV-1 transmission was determined by luciferase activity (RLU) in TZM-bl cell line. Circles represent n = 2 individual donors.

Minor comments:

1. Line 35: The authors state that their findings "demonstrate the pivotal role of intraepithelial multivesicular endosomes as niches for virulent HIV-1". The authors similarly allude to epithelial cells as a viral reservoir in Lines 265-268. It is this reviewer's understanding that epithelial cells and endosomes are short-lived - could the authors comment on the longevity of epithelial cells/epithelial-endosomes such that they might contribute to the HIV reservoir?

- We have observed a LBPA+ intravesicular sequestration of infectious HIV-1 within intestinal epithelial cells for up to 48h (Revised manuscript: Figure 6B,CD and Supplementary Figure S5). The life-span of intestinal epithelial cells ranges between 3-5 days (PMID: 27232601) and epithelial endosomes can be released extracellularly as a result of for example from dying cells (PMID: 38424607), thereby contributing to viral propagation across the intestinal epithelial barrier with implications in the establishment of viral reservoirs within mucosal T-cell and myeloid cell compartments. We have adjusted the revised manuscript accordingly (Revised manuscript: lines 290-296; 317-321).

2. Line 47: The authors state that "we have uncovered that HIV-1 specifically hijacks vesicles studded with a particular molecule (LBPA)". Though this may be true, the authors do not address whether endosomal reorganization within epithelial cells is directly mediated by a viral component (hijacking) versus an anti-viral response by epithelial cells that is indirectly beneficial to the virus.

- We have adjusted the revised manuscript accordingly (Revised manuscript: lines 288-290).

3. Lines 144-146 and Figure 2F - this figure/result is puzzling. The figure legend states "HIV-1 infection of basolateral DCs as a percentage of CD11c+p24+ detached cells, determined by flow cytometer". Were 40% of CD11c+ cells p24+ or did the authors confirm infection in CD11c+p24+ cells by another manner. Further, 40% "infection" seems abnormally high. Is this true infection or sampling - do the authors have data to confirm infection?

- Susceptibility to HIV-1 infection was determined by intracellular p24 staining by flow cytometry analyses in combination with canonical DC markers (CD11c, DC-SIGN) as previously described (PMID: 28024153, 27919079, 33637808). The infection rate of basolateral detached DCs ranged between 4-36% and the suppressive effect of antiretroviral drugs confirmed the productive HIV-1 infection of DCs. Furthermore, co-culture of basolateral detached DCs derived from HIV-1 exposed IEDC co-cultures with permissive cell line resulted in cell-cell HIV-1 transmission to U87.CD4.CCR5 cells, further underscoring the productive HIV-1 infection of the basolateral detached $CD45^{+}CD11c^{+}DC\text{-}SIGN^{+}$DCs. We have included these new data (Revised manuscript: Figure 3H,I, Supplementary Figure S2G and Supplementary Figure S3A,B,C,D) and clarified the findings in the revised manuscript (Revised manuscript: lines 175-185; 517-531; 834-843; 937-941; 950-954)

4. Figure S2B: the empty insert control (dashed line) for the FD4 permeation rate is far below what is shown for other figures and does not match with what is written within the figure legend.

- We apologize for the confusion. In the Figure S2B (*initial version of the manuscript*) we have indeed mistakenly referred to the FD4 permeation rate measured in Figure 3B (*initial version of the manuscript*). For clarity, we have now included per figure all the data points +/- SD obtained for the FD4 permeation rates for the empty insert control in parallel to the FD4 permeation rates obtained for IE cultures. In addition, the average of the FD4 permeation rate from empty insert control was included as a dashed line in each respective figure. We have adjusted the figures (Revised manuscript: Figure 5B, Supplementary Figure S4B) and manuscript accordingly (Revised manuscript: lines 863-869; 963-967).

5. Figure 4C-D/Lines 187-190: It is somewhat unclear what is being demonstrated/discussed. What are the distances shown in Figure 4C? Are these a linear distance from a central point or the average at a given radius?

- Colocalization of HIV-1 with specialized endocytic vesicles was determined by assessing the degree of overlap between HIV-1 and endocytic vesicle fluorescence intensities along the indicated 6 micron-long Region of Interest (ROI) in combination with Pearson's correlation coefficient, in line with previous reports (PMID: 28024153, 27919079, 23719817). We have adjusted the manuscript accordingly (Revised manuscript, lines 563-564, 884-887).

6. Figure 5/Lines 194-200: It was unclear from the text and the figure what was being discuss/shown – clarity was only found within the methods.

- We have adjusted the schematic representation and the revised manuscript accordingly (Revised manuscript: Figure 2A and lines 124-127; 804-819).

7. Although the authors note that their sorting procedures have historically produced high purity populations, the authors did not comment on the purity of their current preparations. The manuscript would be stronger were they to include this information.

- As requested, we have now included the extended phenotypic characterization used in this study depicting the isolation of highly pure monocyte population (~96% $CD45^+CD14^+$ monocytes) and prototypical monocyte-derived DCs (~90% $CD45^+CD11c^+$DC-SIGN$^+$ DCs). We have included these data (Revised manuscript: Supplementary Figure S2A,B,C) and clarified the findings in the revised manuscript (Revised manuscript: lines 393-401 and 917-927)

8. The authors spend considerable effort describing their 2D IEDC model at the beginning of the paper and in Figure 2 but do not refer to it again until Figure 5. To avoid confusion by the reader, it may be more useful to describe the epithelial-centric findings in the beginning of the manuscript and to develop/utilize the IEDC model toward the end as a biologically relevant model.

- We thank the reviewer for the suggestion. In the revised manuscript, the order as well as the content of the main figures and supplementary figures have been considerably adjusted with inclusion of novel experimental data, novel figures and additional datasets as per reviewers' comments. It is the authors' opinion that this revised data presentation endorses the readability of our manuscript.

Response to reviewer 2

Summary: In this article, Rader et al use an in vitro primary intestinal epithelial culture system to examine the interaction of these cells with HIV-1 and Mo-derived DCs. These interactions have relevance to mucosal HIV-1 transmission and likely can only be studied on a mechanistic level through these in vitro epithelial monolayer systems. There are limitations to this system that are inherent drawbacks for any study employing this model, such as the lack of mucus, commensal microbes, and resident immune cells which are all indispensable components of the gut epithelium. The system is further complicated by the fact that the authors inoculate the culture with HIV at very high titers, which is likely not relevant for early events of transmission. The strength of this article is the author's finding that epithelial uptake of HIV can be blocked in vitro by an LBPA antibody, which to my knowledge is novel. There are certain aspects of these experiments however that within this in vitro model, the authors could have employed designs that are more physiologically relevant. Notably by testing direct DC cell-to-cell transmission to targets rather than the cell-free supernatant used in Figure 2. I also am very surprised at the very high level of p24 staining in DCs. The authors do not provide representative flow dot plots as they show for the HIV permissive cell line (which are

surprisingly much lower for p24 than DC). Lastly, there are some statements on extending this to HIV persistence throughout the discussion that should be tempered, as this system is at most a 17 day in vitro culture.

Major comments:

1. Figure 1E. It is not clear at what point after in vitro culture of epithelial cells the authors test HIV-1 transmission of U87 cells across the monolayer. This is important because the epithelial layer does not appear to gain integrity until D14 (Fig 1C.D), and what the authors could me measuring may be passive diffusion of HIV.

- We apologize for the apparent confusion. In concordance to previous reports (PMID: 36951188, 32656095), the experimental procedures employed in our manuscript to generate IE cultures ensures the establishment of polarized, tight and non-permeable monolayers prior to HIV-1 infection. Only IE cultures deemed polarized (by confocal imaging), tight (by longitudinal trans-epithelial electrical resistance measurements) and non-permeable (by fluorescein isothiocyanate-conjugated dextran 4kDa permeation rates from apical to basolateral compartments) on day 14 were subsequently exposed to HIV-1. We clarified the methodology (Revised manuscript: lines 112-120) and further adjusted the schematic representation (Revised manuscript: Figure 1A). Furthermore, we have demonstrated that passive diffusion mechanism does not underlie the observed HIV-1 transmission by IE cultures as evident by the non-paracellular permeability rates of IE cultures before and after inoculation with multiple HIV-1 strains (Revised manuscript: Figure 5B). In fact, our data rather revealed that a LBPA-dependent endosomal trafficking pathway supports the epithelial cell-mediated HIV-1 transmission across intact polarized IE cultures (Revised manuscript: Figures 6A,B,C, 7A,B,C,D,E,F).

2. Figure 2F. It is peculiar that p24 expression in detached primary CD11c+ DCs is much higher (60% in some samples) than in a CCR5-expressing permissive cell line (Figure 1G, H). The primary DCs are likely not productively infected and I am skeptical that gag would be amplified to a degree that would permit such high p24 detection in the DCs. How was the p24 gate set for figure 1F?? The authors only show CD11c gating. The authors need to show representative flow dot plots of DC p24 expression plus or minus HIV.

- For clarity, Figure 2F (*initial version of the manuscript*) depicted the rate of HIV-1 infection of basolateral detached CD11c$^+$ DCs derived from virus-exposed IEDC co-cultures, whereas Figure 2G,H (*initial version of the manuscript*) depicted the rate of cell-free HIV-1 transmission by epithelial cell-derived basolateral medium to U87.CD4.CCR5 cell line. As requested, we now show the infection rates of direct HIV-1 infection of U87.CD4.CCR5 cells and monocyte-derived DCs. Indeed, both U87.CD4.CCR5 cell line (~80% p24$^+$cells) and DCs (10-26% p24$^+$cells) are permissive to HIV-1 infection and cART drugs block HIV-1 infection in either cell type, confirming productive HIV-1 infection of these target cells. Additionally and in line with previous studies (PMID: 27919079, 33637808), we included the gating strategy to determine HIV-1 infection using flow cytometer analyses depicting the absence of HIV-1 p24+ cells in the gated CD11c$^+$DC-SIGN$^+$DCs derived from non-infected IEDC co-cultures *versus* HIV-1 p24+ cells in the gated CD11c$^+$DC-SIGN$^+$DCs in the virus-exposed IEDC co-cultures. We have included these additional data (Revised manuscript: Supplementary Figure S1A, Supplementary Figure S2G and Supplementary Figure S3A) and clarified the findings in the revised manuscript (lines 160-165; 175-185; 393-401; 910-916; 937-941; 943-947)

3. Does HIV activate the detatched dendritic cells by CD80/86 expression as the authors show for LPS in supplemental? This is again a confirmation of the very high p24 expression in DC the authors are showing in Figure 2F.

- As requested, we now show that HIV-1 infection results in DC activation as evident from upregulation of activation marker CD83 and to a lesser degree CD86 in basolateral detached DCs. We have included these data (Revised manuscript: Figure 3G) and clarified the findings in the revised manuscript (lines 178-180; 840-843).

4. Figure 2G, H. It is unclear why the authors chose to culture target cells with IEDC supernatant and not the DC's themselves given that the authors have demonstrated very high intracellular p24 in the DCs (Figure 2F). The latter would mimic cell-to-cell spread which is likely the more physiologically-relevant scenario.

- As requested, we now show that co-culture of basolateral detached DC derived from virus-exposed IEDC co-cultures with target cell line U87.CD4.CCR5, resulted in HIV-1 transmission to the U87.CD4.CCR5 cells through cell-cell contact, which further underscores the productive HIV-1 infection of basolateral detached $CD11c^{+}$DCs. We have included these new data (Revised manuscript: Supplementary Figure S3B,C,D) and clarified the findings in the revised manuscript (lines 182-185; 533-540; 934-843).

5. Line 164-165. "Together, these findings indicate that apical exposure of intact intestinal epithelium to HIV-1 does not readily compromise tight junction function nor barrier permeability." The physiological significance of this finding is unclear as the authors are using a system devoid of critical cellular/microbial components of the intestinal epithelium. In vivo, damage to these components (ie, CD4 T cells) by HIV or amplification of pro-inflammatory cytokines by resident innate immune cells most certainly compromises the epithelium.

- We have adjusted the revised manuscript accordingly (lines 212-215; 279-282).

6. Figure 4. A key finding of this work is localization of HIV to LBPA-containing vesicles. Many readers while versed in HIV-1 immunology/pathogenesis may be naïve to the basic biology of endosomal trafficking in epithelial cells. It would help, preferably in the intro or as a preface to Figure 4 to provide background on the underlying biology of all the endosomal markers and their significance.

- We thank the reviewer for the suggestion and we have adjusted the revised manuscript accordingly (Revised manuscript: lines 225-230).

7. Line 228-229: "For example, in both in vitro and in vivo settings, lung bronchial epithelium harbors latently infected cells, which can be reactivated and resume HIV-1 replication upon inflammation." I would be extremely careful about this statement. The paper the authors are citing (ref 39) only showed vDNA in an in vitro epithelial cell system but to my knowledge no study has shown integrated HIV DNA in epithelial cells of PLWH.

- We thank the reviewer for pointing this out. We have now focused on the in vitro lung bronchial epithelial cells and adjusted the revised manuscripts accordingly (revised manuscript lines 270-272).

8. Line 241-243. "The lasting sequestration of HIV-1 within LBPA-containing vesicles may have additional implications beyond those showcased in the current study, including establishment of latent tissue viral reservoirs and HIV-1 associated immune activation." The authors again need to be very careful here as their own data only shows sequestration of HIV by epithelial cells for 72 hours and extending this to latent tissue reservoirs in PWH, which persist for years/decades, is overzealous in my opinion.

- As requested, we have adjusted the revised manuscript by further outlining the potential implications of LBPA-mediated transcellular transport across the intestinal epithelial barrier in the

establishment of viral reservoirs within the subepithelial mucosal T cell and myeloid cell compartment (Revised manuscript, lines 288-296).

Minor comments:

1. Line 240. Minor comment. Pro-viral could easily be interpreted as proviral 'integrated DNA', this is not what the authors are referring to here.

- As suggested, we have adjusted the revised manuscript accordingly (line 291).

Response to reviewer 3

Summary: The current manuscript by Rader and colleagues titled "HIV-1 exploits LBPA-dependent intraepithelial trafficking for productive infection of human intestinal mucosa" describes a 2D primary intestinal epithelium model, derived from human 3D organoids, for studying mechanisms of enteric viral entry. The authors here show a human intestinal immune-organoid HIV-1 infection using polarized intestinal epithelium and human DCs. The model is useful for studies on viral infection mechanisms in vitro and antiviral drug screening. Data shows when using multiple donors, incubation with epithelial cell-derived basolateral medium resulted in the productive HIV-1 infection of U87.CD4.CCR5 target cells. Human intestinal epithelium-DC (IEDC) co-cultures were established via sequential seeding of primary intestinal epithelial stem cells followed by primary DCs onto a permeable membrane (3 micron inserts). They found that junctional complex expression patterns at cell-cell borders (i.e., ZO-1), and adherence junctions (e-cadherin) remained unaltered upon HIV-1 inoculation, in contrast to the positive control of TNF-α-induced disruption of intestinal epithelial integrity. Other experiments also concluded that HIV-1 follows a specialized LBPA-dependent transcellular trafficking route within human intestinal epithelium. Overall, I found the paper very novel and potentially significant in generating reagents that other colleagues in the field can also use for detailed viral spread and ART studies in cells other than T-cells.

Major comments:

1. Although the authors have cited few papers on HIV-1 penetration of renal, tonsil, foreskin, and cervical epithelial cells (37, 40), they have not cited some of the pioneering ex-plant work from Leonid Margolis lab (NIH) and some of the recent 3D CNS and HIV infection papers.

- As requested, we have now included additional relevant literature and adjusted the revised manuscript accordingly (reference #56-59, lines 271-273).

2. The virology portion of the paper is rather weak and it could use multiple viral isolates (R5, X4) and some clinical isolates to further validate and also add value to the paper.

- As requested, we now show that CCR5-tropic HIV-1 variants (including lab-adapted NL4.3BaL as well as transmitted/founder HIV-1 strains CHO58 and THRO), and to lesser extent CXCR4-tropic HIV-1 variant NL4.3, are transmitted across IEDC co-cultures. These novel data further highlights the biological value of the manuscript and of the established immune-organoid model to study selective intestinal HIV-1 transmission. We have included these novel data (Revised manuscript: Figure 4, Figure 5B) and clarified the findings in the revised manuscript (lines 192-198; 212-215; 851-857; 863-869)

3. The cART portion of the paper is also weak and could use titrations and also different combinations of entry and exit inhibitors to at least solidify the receptor mediate entry of the virus.

- As requested, we now show that prophylactic treatment with nucleotide analog reverse transcriptase inhibitor Tenofovir (TFV), even at wide-concentration range (6.25, 12.5, 25, 50 µM), did

not limit epithelial-mediated HIV-1 transmission. Furthermore, epithelial cell-mediated HIV-1 transmission remained impervious to prophylactic treatment with different classes of cART drugs including CCR5 antagonist Maraviroc (MCV), nucleoside analog reverse transcriptase inhibitor Emtricitabine (FTC) and protease inhibitor Indinavir (IDV). Additionally, two-drug combinatory treatments of TFV with either FTC or IDV did not reduce HIV-1 transmission by epithelial cells. These additional data thus further corroborate that different classes or regimens of direct-acting antivirals are not suitable in suppressing HIV-1 transmission by intestinal-derived epithelial cells *ex vivo*. We have included these new data (Revised manuscript: Figure 2 A,B,C,D,E,G,H) and clarified the findings in the revised manuscript (lines 122-146, 465-481; 804-819).

Minor comment:

1. Although the authors have cited few papers on HIV-1 penetration of renal, tonsil, foreskin, and cervical epithelial cells (37, 40), they have not cited some of the pioneering ex-plant work from Leonid Margolis lab (NIH) and some of the recent 3D CNS and HIV infection papers.

- We have adjusted the manuscript accordingly (reference #56-59, lines 271-273).
