# Supplementary material for: HIV-1 exploits LBPA-dependent intraepithelial trafficking for productive infection of human intestinal mucosa
Source: PLoS Pathog. 2024 Dec 27;20(12):e1012714. doi: 10.1371/journal.ppat.1012714 (PMC11676502; doi:10.1371/journal.ppat.1012714)
Supplement: Attachment — Submitted filename: Rader et al. response to reviewer 2.pdf [file ppat.1012714.s008.pdf]

Response to reviewer 2

Summary: This manuscript is much improved. I just have a minor critique on the new supplemental 3C.

Minor Issue:

1. I would recommend that in the revised supplemental figure 3C for viral cell-cell transmission, the authors denote in the figure the 'cd11c-' population as actually representing the U87.cd4.ccr5 permissive cell line for clarity. To the reviewer it was easily mis-interpreted as the "CD11c-negative non-DC fraction" in revised fig 3H,I, which the authors show to not express any p24.

- As requested, we have adjusted accordingly the figure and labelled the population as "CD11c- U87.CD4.CCR5" to clearly denote the HIV infection of U87.CD4.CCR5 in the cell-cell transmission setting (Revised manuscript: Supplementary Figure 3C).
